# Pelargonidin Inhibits Isoproterenol Induced Myocardial Fibrosis via Regulating Transforming Growth Factor-β/Smad2/3 Signaling and Th2 Cytokines in Mice

**DOI:** 10.33549/physiolres.935673

**Published:** 2026-02-01

**Authors:** Juanhua ZHAO, Linping DONG, Long YAO

**Affiliations:** 1 The Seventh Department of Cardiovascular Medicine, Xi’an International Medical Center Hospital, Xi’an, China; 2 Department of Cardiology, Shandong Second Provincial General Hospital, Jinan, China; 3 Department of Cardiology, Yan’an People’s Hospital, Yan’an, China

**Keywords:** Pelargonidin, Myocardial fibrosis, Transforming Growth Factor-β, Extracellular matrix, Cytokines

## Abstract

Pelargonidin, a natural anthocyanidin, is known for its anti-inflammatory, antioxidant, and cytoprotective properties, however its role in myocardial fibrosis remains unclear. This study explored the therapeutic potential of pelargonidin in a mouse model of isoproterenol (ISO)-induced myocardial fibrosis. Male C57BL/6 mice were treated with ISO and subsequently administered either a low (20 mg/kg/day) or high (40 mg/kg/day) dose of pelargonidin, with captopril (15 mg/kg/day) serving as a reference control. Histological analysis revealed that pelargonidin significantly reduced collagen deposition in the myocardium in a dose-dependent manner. Molecular assessments showed decreased protein expression of α-SMA, COL3A1, and FN1, along with downregulation of TGF-β/Smad2/3 signaling, as evidenced by reduced levels of TGF-β and phosphorylated Smad2/3. Additionally, pelargonidin suppressed the expression of extracellular matrix-related genes and decreased circulating levels of Th2 cytokines IL-4 and IL-13. These findings indicate that pelargonidin mitigates myocardial fibrosis by targeting the TGF-β/Smad2/3 pathway and modulating Th2-mediated immune responses. Overall, the results suggest that pelargonidin may serve as a promising therapeutic agent for myocardial fibrosis and related cardiovascular disorders.

## Introduction

Heart failure (HF) is an increasingly prevalent public health concern, affecting an estimated 64 million individuals worldwide [1]. Chronic heart failure (CHF) is a multifactorial and complex clinical condition that arises from various underlying cardiovascular disorders [2]. CHF is associated with significant alterations in the structure and function of the heart [3]. Epidemiological studies show that in developed countries the prevalence of CHF is between 1 to 2 %, with a sharp increase in older populations, making it a major public health concern [4]. The development and progression of CHF leads to myocardial fibrosis (MF) and is perhaps the most central pathological process of CHF [5,6]. Fibrotic remodeling increases myocardial stiffness, reduces compliance and contractility, and thereby exacerbates heart failure [7]. Emerging evidence highlights the critical role of immune-inflammatory activation in CHF, as pro-inflammatory cytokines, immune cell infiltration, and maladaptive immune responses contribute to the initiation and progression of myocardial fibrosis, ultimately impairing cardiac function. Understanding these pathways has prompted the exploration of immunomodulatory and anti-inflammatory therapies as potential strategies to attenuate fibrosis and improve outcomes in CHF.

Anti-inflammatory and immunomodulatory therapies have recently gained considerable attention as potential interventions for congestive heart failure (CHF) [8]. Preclinical studies demonstrate that inflammatory modulators such as methotrexate, rapamycin, and triptolide can improve cardiac function and reduce fibrosis in animal models [9,10]. These findings have spurred global efforts by clinicians and researchers to develop effective treatment strategies for CHF. This growing recognition that inflammation is a modifiable target has shifted the focus toward exploring both natural and synthetic anti-inflammatory agents, including pelargonidin, as promising therapeutic candidates.

In recent years, numerous studies have demonstrated the potential of herbal products as alternative therapeutic regimens for various clinical conditions [11]. Understanding the molecular events and key mediators in myocardial fibrosis pathogenesis would be valuable in the development and identification of novel therapeutic agents with higher efficiency and negligible adverse reactions. Anthocyanins and anthocyanidins have been reported to provide multiple health benefits, including antioxidant, antitumor, neuroprotective and anti-diabetic properties *in vivo* [12]. Pelargonidin and its glucoside, pelargonidin-3-glucoside found in berries, grapes, red radish, and other vegetables and fruits have been reported to possess pharmacological properties as anti-inflammatory, antioxidant and cytoprotective effects [13]. Pelargonidin, a major anthocyanidin, has shown potent antioxidant and anti-inflammatory activities by scavenging reactive oxygen species (ROS) and inhibiting pro-inflammatory mediators such as TNF-α, IL-1β, and IL-6 [13]. Mechanistically, Anthocyanins has been reported to suppress NF-κB activation, a key regulator of inflammation, and to modulate MAPK signaling pathways, thereby reducing cytokine-driven tissue damage [14]. Pelargonidin-3-O-glucoside (Pg3G) administration prevented ISO-induced elevations of cardiac injury markers (CK, CK-MB, cTnT, cTnI), reduced pro-inflammatory cytokines (IL-6, IL-1β, TNF-α), and restored antioxidant defenses (GSH, GPx, CAT, SOD). Gene expression analyses further confirmed its ability to mitigate ISO-induced inflammation, fibrosis, and cardiac toxicity [15]. These actions collectively suggest that pelargonidin may protect against cardiac remodeling and fibrosis in CHF.

TGF-β has a prominent role in the process of fibrogenesis pathology for many organ systems [16). Fibrotic cascade begins with TGF-β binding to its receptors on the fibroblast surface where it starts a Smad protein signaling cascaded, chiefly dominated by Smad2 and Smad3 [17]. TGF-β binding to its receptor induces receptor phosphorylation, which subsequently activates Smad2 and Smad3. These phosphorylated proteins then form a hete-rodimer complex with Smad4 [18]. They migrate into the nucleus, where this heterodimer assembly regulates regulate the expression of genes controlling the extracellular matrix (ECM). Dysregulation of this pathway results in excessive ECM accumulation, leading to tissue stiffening and impaired organ function. In CHF, activation of the TGF-β/Smad2/3 pathway drives myocardial fibrosis, increasing ventricular stiffness and accelerating disease progression [19].

Th2 cytokines such as IL-4 and IL-13 play a crucial role in promoting fibrogenesis by creating a pro-fibrotic microenvironment [20]. Produced by Th2-polarized immune cells, these cytokines stimulate fibroblast activation and their differentiation into myofibroblasts, the primary cells responsible for excessive extracellular matrix (ECM) production. Among them, IL-13 has been shown to synergize with TGF-β, enhancing collagen synthesis and deposition [21]. This interaction establishes a self-sustaining loop, where Th2 cytokines and TGF-β signaling perpetuate chronic inflammation, fibrosis, and progressive tissue damage. While IL-13-driven fibroblast proliferation is well-documented in pulmonary fibrosis, similar mechanisms in CHF contribute to myocardial remodeling, leading to structural and functional deterioration of the heart [22].

Pelargonidin modulates oxidative stress, inflammation, and fibrotic signaling, making it a promising candidate for targeting CHF-related fibrosis. It has demonstrated cardioprotective effects by reducing oxidative damage, suppressing pro-inflammatory cytokines, and improving vascular function. Its ability to inhibit TGF-β signaling and downregulate fibrogenic mediators suggests potential to prevent fibroblast activation and excessive ECM deposition. This study investigates pelargonidin’s therapeutic effects on myocardial fibrosis in a mouse CHF model, focusing on its modulation of the TGF-β/Smad2/3 pathway and Th2 cytokine regulation.

## Materials and Methods

### Kits, reagents and antibodies used

The following materials were used in this study: Isoproterenol (Cat# I6504, Purity: ≥98 %), pelargonidin (Cat# P1659, Purity: ≥90 %) and captopril (Cat# C4042) were obtained from Sigma-Aldrich, St. Louis, MO, USA. LDH assay kit (Cat# ab102526) and CK assay kit (Cat# ab155901) were obtained from Abcam, Cambridge, UK. IL-4 (Cat# 88-7044-88) and IL-13 (Cat# 88-7137-88) ELISA kits were purchased from ThermoFisher Scientific, Waltham, MA, USA. Hydroxyproline assay kit (Cat# MAK008) was procured from Sigma-Aldrich, USA. TRIzol™ reagent (Cat# 15596026) and RevertAid First Strand cDNA Synthesis Kit (Cat# K1622) were obtained from ThermoFisher Scientific, USA. All-in-One™ Quantitative Real-Time PCR Mix (Cat# QP001) was purchased from GeneCopoeia, Rockville, MD, USA. The qPCR was performed using the Applied Biosystems StepOnePlus™ Real-Time PCR System (ThermoFisher Scientific, USA). Bradford protein assay kit (Cat# P0006) was obtained from Beyotime Institute of Biotechnology, Nantong, China. Primary antibodies including TGF-β1 (Cat# 3711), Smad2 (Cat# 3103), phospho-Smad2 (Cat# 3108), Smad3 (Cat# 9523), phospho-Smad3 (Cat# 9520), α-SMA (Cat# 19245), COL3A1 (Cat# 22734), FN1 (Cat# 26836), and β-actin (Cat# 4970) were purchased from Cell Signaling Technology, Danvers, MA, USA. HRP-conjugated secondary antibodies (Cat# 7074 and 7076) were also obtained from Cell Signaling Technology, USA. ECL Western blotting detection reagent (Cat# WBKLS0500) and PVDF membranes (Cat# IPVH00010) were procured from Millipore, USA. The Masson’s Trichrome Staining Kit (Cat# G1340) was obtained from Solarbio Life Sciences, Beijing, China, and the immunohistochemistry staining kit (Cat# ab80436) was sourced from Abcam, UK. Protein quantification was carried out using the BCA Protein Assay Kit (Cat# 23225; ThermoFisher Scientific, USA).

### Animal experiments

Thirty [30] C57BL/6 mice, relatively young (4 to 6 weeks) and weighing (220 to 250 g) were obtained from the Experimental Animal Center of Yan’an People’s Hospital, Yan’an, China. They were randomly divided into 5 groups: a control (n=6), ISO (n=6) (100 mg/kg), ISO + low dose of Pelargonidin (n=6) (20 mg/kg/day), ISO + high dose of Pelargonidin (n=6) (40 mg/kg/day), and ISO + Captopril (n=6) (15 mg/kg/day) groups [23,24,25]. Captopril is widely used as a standard reference drug in studies of myocardial fibrosis and heart failure because of its well-established cardioprotective and anti-fibrotic effects [26]. The control group received subcutaneous normal saline injections for 47 consecutive days. The rest of the mice groups received subcutaneous ISO injections to induce heart failure. The dosing schedule for ISO included an initial dose of 5 mg/kg on the 1^st^ day and a gradual tapering to 2.5 mg/kg for the next 47 days, so the total duration would be 47 days. Pelargonidin was administered *via* oral gavage for 47 days to the treatment group mice starting from 1^st^ day of ISO injection to day 47. The low dose Pelargonidin group received daily doses of Pelargonidin at 20 mg/kg, whereas the high dose Pelargonidin group received daily doses of Pelargonidin at 40 mg/kg. Mice in the Captopril group received Captopril (15 mg/kg) for 47 consecutive days. No deaths were recorded through the duration of the experiment.

Subsequent to the 47-day treatment period, mice were anesthetized using pentobarbital (2 % solution, 0.2 ml/100 g, *via* intraperitoneal injection). The hearts were immediately excised, rinsed in ice-cold PBS, and either stored at −80 °C for biochemical analysis or fixed in 4 % paraformaldehyde for immunohistochemistry.

All procedures involving animals were carried out in compliance with our institutional guidelines for the use of laboratory animals. The current study was approved by the Zhinanzhen Biology Ethics Committee, (No.: A2024000162). After confirming adequate anes-thesia, the hearts were surgically excised with strict measures taken to minimize pain, distress, and suffering throughout the procedure.

### Histopathology

Mice ventricular tissue samples were immediately fixed in 10 % neutral buffered formalin (Sigma-Aldrich) for 24–48 h at room temperature. Following fixation, the tissues were dehydrated through a graded ethanol series, cleared in xylene, and subsequently embedded in paraffin wax using a standard tissue processor. Paraffin blocks were sectioned into 4–5 μm-thick slices using a rotary microtome (Leica RM2235, Germany).

To evaluate myocardial fibrosis, sections were stained using Masson’s trichrome staining kit (Sigma-Aldrich) according to the manufacturer’s protocol. In this staining method, collagen fibers appeared blue, muscle fibers red, and cell nuclei black, allowing clear visualization and quantification of fibrotic areas under a light microscope.

For immunohistochemistry (IHC), sections were first deparaffinized in xylene, rehydrated through graded ethanol, and rinsed in distilled water. Antigen retrieval was performed by heating the sections in citrate buffer (pH 6.0) using a microwave for 10–15 min. After cooling to room temperature, endogenous peroxidase activity was blocked by incubating the sections with 3 % hydrogen peroxide for 10 min. The slides were then blocked with 5 % bovine serum albumin (BSA) for 30 min to prevent nonspecific binding. Next, the sections were incubated overnight at 4 °C with a primary antibody against collagen I (e.g., ab21286, rabbit anti-Collagen I, 1:200 dilution, Abcam). After washing with PBS, the slides were incubated with HRP-conjugated secondary antibody (31460, 1:10000 dilution, ThermoFisher scientific, USA) for 30 min at room temperature. Visualization was achieved using a 3,3′-diami-nobenzidine (DAB) substrate kit (D7304, Sigma-Aldrich), producing a brown precipitate at the site of antigen localization. The sections were then counterstained with hematoxylin, dehydrated, cleared, and mounted with neutral balsam.

Immunopositive staining for collagen I was identified as fine brown granules within the myocardial tissue and examined using a fluorescent microscope (Evos-FL, Thermo Fisher Scientific, USA). Images were captured, and the extent of collagen I expression was quantified using ImageJ software (NIH, USA) for statistical analysis.

### Collagen concentration measurement

A sample of ventricular tissue weighing between 30–100 mg (wet weight) was collected and centrifuged. The tissue was further incubated in 1 ml of hydrolytic reagent and placed in a water bath at 95 °C for 20 min for hydrolysis. After incubation, pH of the solution was adjusted to be in the range of 6.0–6.8. The concentration of hydroxyproline, considered to be a surrogate measure for the amount of collagen present, was measured using commercially available hydroxyproline assay kit following the instructions given in the manual.

Collagen concentration was calculated using the following formula:

Collagen concentration (μg/mg) = Hydroxy-proline concentration × 7.5

The concentration of hydroxyproline in ventricular tissue was determined with the following equation:

Hydroxyproline concentration (μg/mg) = [(Absorbance of sample tube – Absorbance of blank) × standard concentration (5 μg/ml) × Total reaction volume (10 ml)] ÷ [(Absorbance of standard – Absorbance of blank) × weight of wet tissue in mg]

### Western blot analysis

Briefly, heart tissues from mice were homogenized in ice-cold RIPA lysis buffer supplemented with protease and phosphatase inhibitors to extract total proteins. The homogenates were centrifuged at 12,000× g for 15 min at 4 °C, and the supernatants were collected for further analysis. Protein concentrations were determined using the BCA protein assay kit. Protein samples were processed through 8–10 % SDS-PAGE and later on blotted onto PVDF membrane. The membranes were treated with 5 % BSA and incubated overnight at 4 °C with primary antibodies such as, TGF-β1, Smad2, phospho-Smad2, Smad3, phospho-Smad3, α-SMA, COL3A1, FN1, and β-actin. Afterward the members were washed thrice with TBS and Tween 20. The membranes were probed with secondary antibodies, HRP conjugated for two hours at room temperature (RT). Subsequent to additional washes with TBST, immunoreactive bands were identified using ECL Western blotting detection reagent (Millipore) and the bands were visualized on UVP ChemStudio System.

### Real-Time PCR (RT-PCR) analysis

Myocardial tissues were first flash frozen in liquid nitrogen and completely homogenized by using a pre-chilled mortar and pestle. Following this step, 1 ml of TRIzol™ Reagent was added to the homogenized heart tissues and RNA was extracted by following the phase separation and RNA precipitation processes outlined by the manufacturer. The extracted RNA pellets underwent washing with 75 % ethanol, were air-dried, and subsequently resuspended in RNase-free water.

RNA was treated with RNase-free DNase I to prevent persistent genomic DNA contamination. The treatment was performed at 37 °C for half an hour. Concentration and purity of RNA was calculated using a NanoDrop spectrophotometer, measuring the ratios A_260_/A_280_ and A_260_/A_230_. Subsequently, the RNA underwent electrophoresis on a 1 % agarose gel which alongside measuring the aforementioned ratios serves as an additional step to ensure sharp 28S and 18S rRNA bands (cRNA bands) were present.

The first step of the procedure was performed using a reverse transcription kit, specifically employing the RevertAid First Strand cDNA Synthesis Kit. The first order steps include utilization of one microgram of total RNA with oligo(dT) primers and reverse transcriptase leading to cDNA. The cDNA products can either be placed immediately on ice or at −20 °C for extended periods of time.

All-in-One™ Quantitative Real-Time PCR Mix was used for performing qRT-PCR on 2 μl of cDNA in a total reaction volume of 20 μl. Reactions were run on a real-time PCR system with the following settings: initial 10 min denaturation at 95 °C, followed by 40 cycles of 15 s at 95 °C, and 1 min at 60 °C. Matrix metalloproteinase 9 (MMP9), Tissue inhibitor of metalloproteinases-1 (TIMP1), and elastin were selected as target genes for extracellular matrix remodeling. The sequences of the primers are listed in Table 1.

For confirmation of technical accuracy, each qRT-PCR reaction was performed in triplicate. The normalization of the gene expression was done by taking β-actin as the control gene. The relative values for expression of mRNA were determined using the 2^−ΔΔCt^ method. To improve reliability and reproducibility of the data, final expression values presented were averaged from three independent biological replicates.

### ELISA

Levels of IL-4, IL-13, LDH, and CK in myocardial tissue were measured using commercially available ELISA kits (IL-4 and IL-13: Abcam, UK; LDH and CK: Sigma-Aldrich, USA) following the manufacturers’ instructions [27]. Ventricular tissue samples were weighed and homogenized in ice-cold phosphate-buffered saline (PBS, pH 7.4; Gibco, USA) at a ratio of 1:10 w/v (e.g., 100 mg tissue in 1 ml PBS) using a mechanical tissue homogenizer (TissueRuptor II, Qiagen, Germany) under chilled conditions to prevent protein degradation. Homogenates were centrifuged at 5,000× g for 10 min at 4 °C, and the supernatants were collected into pre-chilled microcentrifuge tubes. Total protein concentration was determined using a BCA protein assay kit (Thermo Fisher Scientific, USA) to ensure equal protein loading. For ELISA, supernatants were added to pre-coated 96-well plates, incubated with specific capture and detection antibodies, and visualized using TMB substrate followed by addition of stop solution. Absorbance was measured at 450 nm using a microplate reader (BioTek Synergy HTX, USA). Each sample was assayed in triplicate, and standard curves were used to calculate absolute concentrations, ensuring accuracy, reproducibility, and reliable quanti-fication of cytokines and cardiac injury markers.

### Statistical analysis

The data is expressed as mean ± standard error of the mean (SEM). All the Statistical Analyses were performed using GraphPad Prism 8.0 software (GraphPad Software Inc., San Diego, CA, USA). For more than two groups, we performed one-way analysis of variance (ANOVA) with *post hoc* pairwise comparisons using Tukey’s test. Statistical significance was set at P<0.05.

## Results

### Impact of Pelargonidin on heart weight to body weight (HW/BW) ratio

**Fig. 1:**
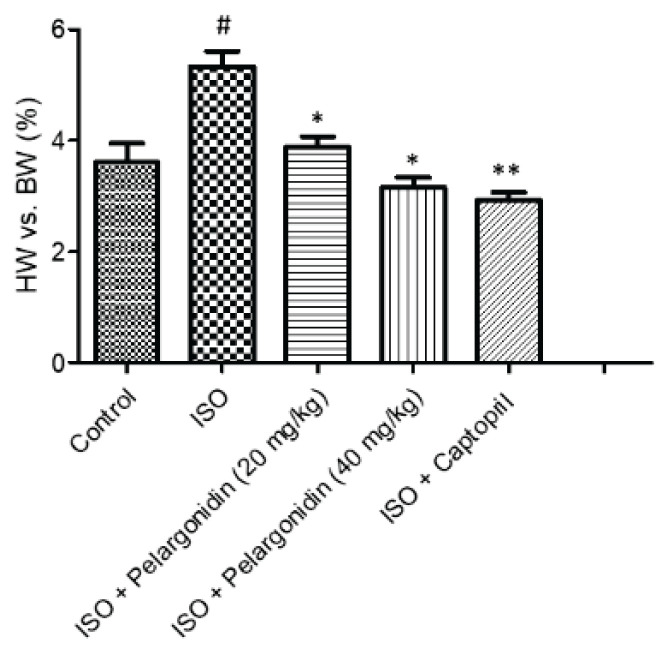
Pelargonidin reduces ISO-induced cardiac hypertrophy in mice. HW/BW ratios were measured after 47 days of treatment. ISO significantly increased HW/BW compared to controls (P=0.02), indicating hypertrophy. Pelargonidin (low: 20 mg/kg/day; high: 40 mg/kg/day) and Captopril (15 mg/kg/day) significantly reduced HW/BW compared to ISO (P=0.03). The ISO + high-dose Pelargonidin (P=0.01) and ISO + Captopril (P=0.007) groups showed greater reduction than ISO + low-dose Pelargonidin. Data are mean ± SEM (n=3). Statistical significance: **^#^** P<0.05 vs. control, * P<0.05, ** P<0.01 vs. ISO.

Upon sacrificing the mice, the heart was removed to weigh it, and the HW/BW ratio was calculated. As illustrated in Figure 1, the ISO group showed a significant increase in the HW/BW ratio as compared to the control group (P=0.02), suggesting the presence of cardiac hypertrophy. Unlike the ISO group, treatment with Pelargonidin (low and high dose) and Captopril showed a significant reduction of this ratio relative to the ISO group (P=0.03) suggesting the reversal of ISO-induced cardiac hypertrophy. Importantly, both ISO + high dose Pelargonidin (P=0.01) and ISO + Captopril groups (P=0.007) showed a greater reduction in HW/BW ratio compared to the ISO + low dose Pelargonidin group.

### Pelargonidin ameliorates cardiac fibrosis and confers cardioprotective effects in vivo

**Fig. 2:**
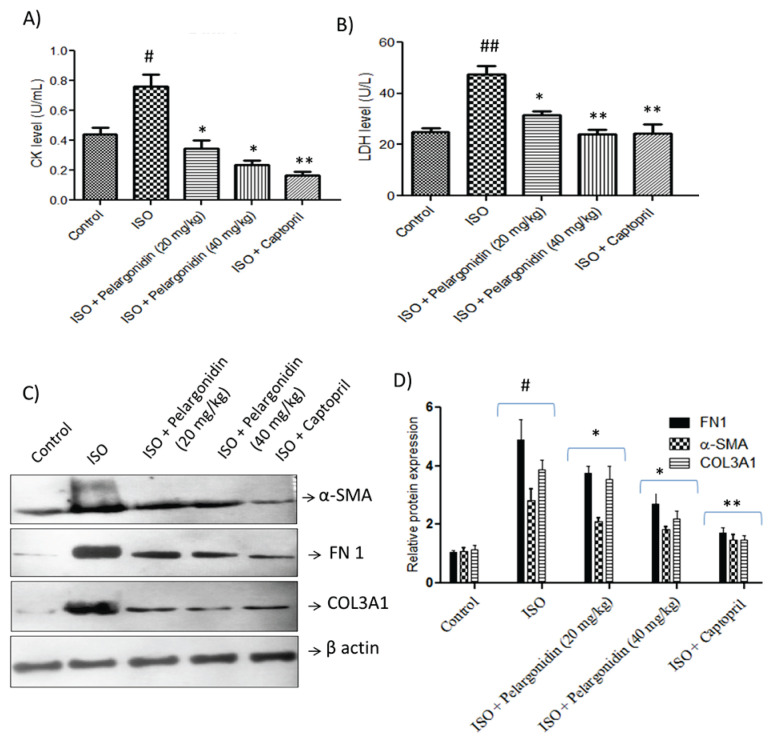
Pelargonidin reduces myocar-dial injury and fibrosis in ISO-induced heart failure. (**A, B**) CK and LDH acti-vity levels were measured in serum to assess myocardial injury. ISO treatment significantly elevated CK and LDH levels compared to controls, indicating cardiac damage. Pelargo-nidin treatment at both low (20 mg/kg/day) and high (40 mg/kg/day) doses significantly reduced these enzyme levels, suggesting cardioprotective effects. (**C, D**) Representative Western blot images and corresponding densito-metric quantification show the expression of fibrosis-related proteins FN1, α-SMA, and COL3A1 in myocardial tissue. ISO treatment markedly increased these proteins, reflecting cardiac fibrosis, while Pelargonidin treatment suppressed their expression in a dose-dependent manner. Data are expressed as mean ± SEM (n=3 per group). Statistical significance was determined by one-way ANOVA followed by Tukey’s *post hoc* test. **^#^** P<0.05, **^##^** P<0.01, vs. control group, * P<0.01, ** P<0.001 vs. ISO group.

In this study, we aimed to assess the therapeutic effects of Pelargonidin on ISO-induced cardiac fibrosis by administering different doses of Pelargonidin to mice with ISO-induced heart failure (HF). It was noted that ISO administration significantly enhanced the LDH and CK levels, which indicates myocardial injury. Treatment with low dose Pelargonidin (20 mg/kg/day) and high dose Pelargonidin (40 mg/kg/day) resulted in significant reduction in LDH levels, as well as CK levels (Fig. 2A, B). This suggests that Pelargonidin may protect against ISO-induced damage to cardiac tissues.

As a result of ISO induction, severe cardiac fibrosis was noted, as seen with the significant upregulation of fibrosis associated proteins α-SMA, COL3A1 and FN1 (Fig. 1C). In the low dose group, Pelargonidin significantly reduced the expression of α-SMA, COL3A1 and FN1, which resulted in even lower reductions for the high dosed group. This proves that Pelargonidin effectively reduces cardiac fibrosis associated with ISO-induced heart failure in mice.

### Pelargonidin reduces collagen deposition

**Fig. 3:**
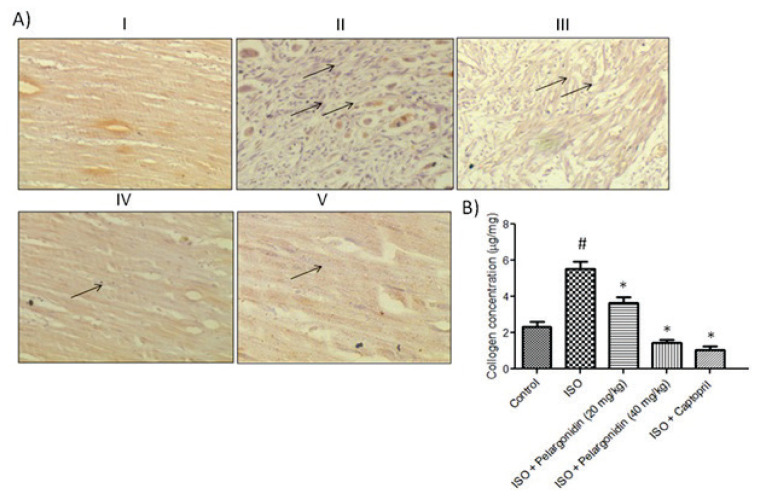
Pelargonidin attenuates collagen deposition in ISO-induced heart failure. (**A**) Representative immunohistochemistry images (40×) of left ventricular tissue illustrating collagen I expression across experimental groups: (I) Control, showing minimal collagen deposition; (II) ISO-induced CHF, exhibiting marked collagen accumulation indicative of myocardial fibrosis; (III) Low-dose Pelargonidin (20 mg/kg/day), showing partial reduction of collagen deposition; (IV) High-dose Pelargonidin (40 mg/kg/day), showing greater reduction; (V) Captopril (15 mg/kg/day) as a positive control, demonstrating reduced collagen levels. (**B**) Quantitative assessment of total collagen content was performed using a hydroxyproline assay, confirming histological findings. Data are presented as mean ± SEM (n=3). Statistical significance was determined using one-way ANOVA followed by Tukey’s *post hoc* test: **^#^** P<0.05 vs. control group, * P<0.05, vs. ISO group.

Collagen accumulation was markedly heightened in the setting of ISO-induced chronic heart failure (CHF), reflecting considerable myocardial fibrosis. In the collagen I control group (Fig. 3A), expression was almost absent; however, ISO treatment showed marked increase underscoring extensive fibrotic remodeling. Low and high dose Pelargonidin treatment clearly diminished this increase in a dose dependent manner, as noted through collagen I staining in the myocardial tissue. These histological observations were complemented with qualitative biochemical assessments. In particular, cardiac hydroxyproline concentration (an indicator of total collagen content) was found to be increased in the ISO group when compared to the control group (P=0.03), validating the hypothesis of collagen deposition due to myocardial fibrosis (Fig. 3B). More importantly, low and high doses of Pelargonidin treatment significantly reduced hydroxyproline concentration when compared to the ISO group (P=0.04 and P=0.03, respectively), suggesting that Pelargonidin possesses the ability to reduce deposition of collagen and advancement of fibrosis. The findings imply that the antifibrotic action of Pelargonidin helps to shield the myocardium from pathologic stress which may justify its effect in heart failure under ISO stress conditions.

### Pelargonidin is associated with TGF-β1/Smad signaling pathway modulation

**Fig. 4:**
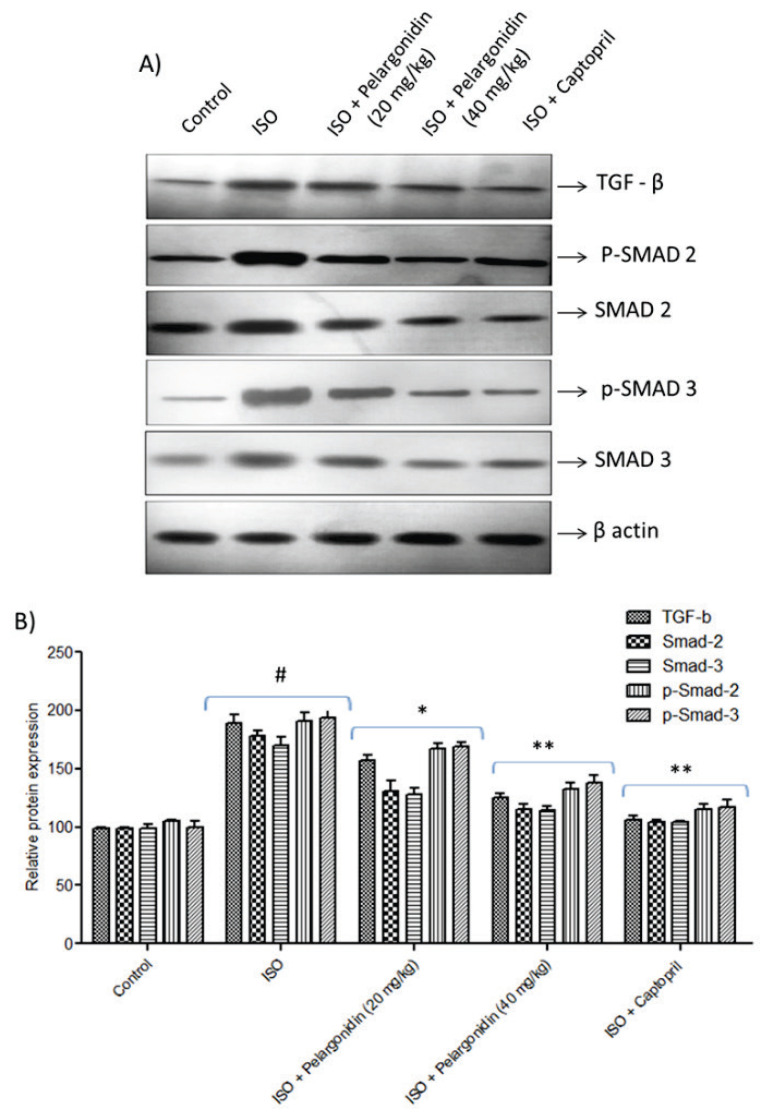
Pelargonidin suppresses TGF-β1/Smad signaling in ISO-induced heart failure. (**A**) Representative Western blot images showing expression of TGF-β1, Smad2, Smad3, p-Smad2, and p-Smad3 in left ventricular tissue across experimental groups. (**B**) Densitometric quantification of the blots confirming the effects of Pelargonidin on the Smad signaling pathway. Data are expressed as mean ± SEM (n=6 per group). Statistical significance was determined using one-way ANOVA followed by Tukey’s *post hoc* test: **^#^** P<0.05, vs. control group, * P<0.01, ** P<0.001 vs. ISO group.

Next, we investigated the possible molecular pathways by which Pelargonidin exerts antifibrotic effects and emphasized the TGF-β1/Smad signaling pathway considering its significance in cardiac fibrotic remodeling. Mice with ISO-induced heart failure showed marked upregulation of TGF-β1 expression with increased phosphorylated Smad2 and Smad3 (p-Smad2 and p-Smad3) along with Smad2 and Smad3 proteins (Fig. 4A, B). These alterations suggest substantial upregulation of the canonical Smad signaling system tends to get activated following stress induced by ISO.

Pelargonidin was found to markedly down-regulate TGF-β1 and the level of p-Smad2 and p-Smad3 which was indicative of downstream fibrotic signaling blockade. Moreover, it was also observed that Pelargonidin helped alleviate the overexpression of Smad2 and Smad3 proteins, which were upregulated in the ISO group. The data indicates that Pelargonidin is able to lessen cardiac fibrosis potentially by altering the TGF-β1/Smad pathway, which provides an explanation for the diminished profibrotic signaling and enhanced myocardial function restoration.

### Pelargonidin modulates mRNA expression of ECM associated genes

**Fig. 5:**
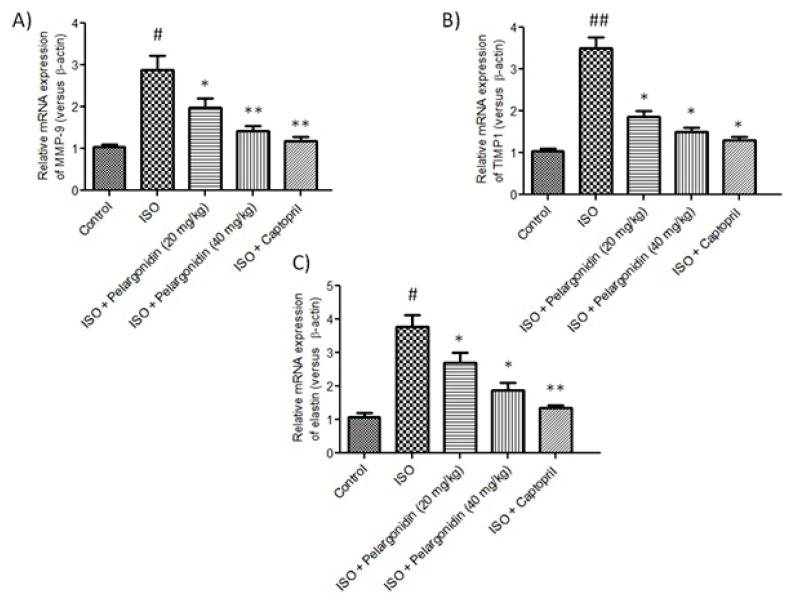
Pelargonidin regulates mRNA expression of ECM-associated genes in ISO-induced heart failure. Quantitative PCR analysis of left ventricular tissue showing relative mRNA expression of MMP-9 (**A**), TIMP1 (**B**), and elastin (**C**) across experimental groups. ISO treatment significantly increased expression of these genes compared to controls, while Pelargonidin treatment dose-dependently suppressed their expression. Data are expressed as mean ± SEM (n=3). Statistical significance was determined using one-way ANOVA followed by Tukey’s *post hoc* test: **^#^** P<0.05, **^##^** P<0.01, vs. control group, * P<0.01, ** P<0.001 vs. ISO group.

Cardiac fibrosis due to heart failure is characterized by accumulation of distinct ECM components along with an imbalance within the ECM, eventually leading to increased stiffness of the heart muscle. To analyze the impact of Pelargonidin on ECM remodeling, it’s needed to determine the mRNA expression levels of relevant genes in ISO-induced heart failure models. Administration of ISO resulted in a considerably higher expression of ECM-associated genes such as MMP-9, TIMP1, and elastin compared to the control group (P<0.05) (Fig. 5A, B and C).

In contrast, Pelargonidin treatment on ISO induced mice has been shown to control ECM expression in a dose responsive manner. Pelargonidin profoundly suppressed the expression of MMP-9 and TIMP1, restoring the equilibrium of ECM degradation and deposition. In addition, expression level of structural ECM protein elastin was significantly lower after treatment with Pelargonidin (P=0.01), suggesting reduction of ECM fibrotic accumulation.

The findings support the notion that Pelargonidin mitigates the expression of fibrotic markers at the transcriptional level while simultaneously restoring ECM equilibrium in the failing myocardium. This shift in the ECM control may be one of the protective mechanisms Pelargonidin employs to counteract ISO-induced cardiac remodeling and fibrosis.

### Pelargonidin modulates Th2 inflammatory markers

To evaluate the contribution of inflammation on ISO-induced cardiac pathology and the immunomodulatory actions of Pelargonidin, IL-4 and IL-13 levels were analyzed in myocardial tissues.

**Fig. 6:**
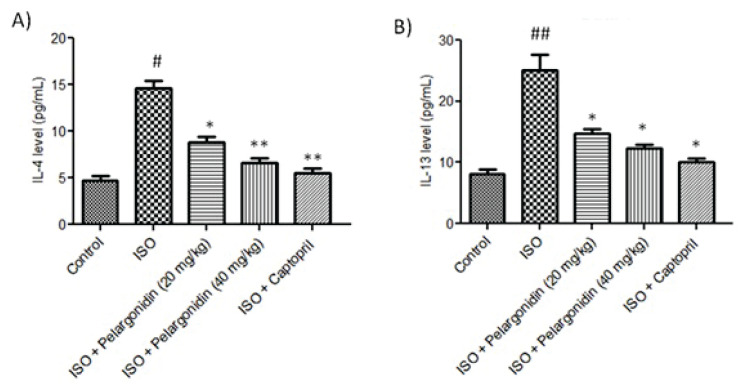
Pelargonidin reduces Th2 inflammatory cytokines in ISO-induced heart failure. Quantification of myocardial IL-4 (**A**) and IL-13 (**B**) levels in different experimental groups. ISO treatment significantly increased cytokine expression compared to controls, while Pelargonidin at both low (20 mg/kg/day) and high (40 mg/kg/day) doses dose-dependently suppressed IL-4 and IL-13 levels. Data are presented as mean ± SEM (n=3). Statistical significance was determined using one-way ANOVA followed by Tukey’s *post hoc* test: **^#^** P<0.05, **^##^** P<0.01, vs. control group, * P<0.01, ** P<0.001 vs. ISO group.

In the control group, IL-4 and IL-13 levels stayed at a minimum baseline range, which indicates normal immunological balance regarding the tissue (Fig. 6A, B). Nevertheless, after administration of ISO, a marked increase in both cytokines was noted this suggests the activation of a Th2-type inflammatory response that is usually linked with chronic cardiac injury and fibrotic remodeling.

Moreover, treatment with Pelargonidin at 20 mg/kg/day had significantly lower IL-4 and IL-13 levels than the ISO group (P=0.03) resulting in attenu-ation of ISO-induced inflammation. Also, administration of Pelargonidin at 40 mg/kg/day had a further increase in the reduction of these cytokines, approaching the levels seen in control groups. This clear dose-dependent reduction illustrates the anti-inflammatory role of Pelargonidin in ISO induced heart failure.

## Discussion

Myocardial fibrosis (MF) represents one of the primary pathological processes that drive heart failure and is noted for the damage caused due to excessive deposition of other ECM components like collagen [28]. In this study, we present novel findings of the significant cardioprotective effect exhibited by Pelargonidin, an anthocyanidin existing in several fruits and vegetables, on ISO-induced myocardial fibrosis in a murine model. We present data that Pelargonidin mitigates myocardial fibrosis by acting on the TGF-β/Smad2/3 signaling mechanism and subsequently on the Th2 response cytokines, notably IL-4 and IL-13.

The isoproterenol (ISO) model has gained vast popularity when it comes to studying stress-induced cardiac injury and fibrosis because of its simple execution and reproducibility [29]. ISO administration causes sustained β-adrenergic activation, oxidative stress, inflammation, fibrotic remodeling, and necrotic tissue buildup, leading to myocardial inflammation, necrosis, and fibrosis. In our experiments, myocarditis induced through ISO administration resulted in substantial increments in serum LDH and CK values, indicative of cardiac myocyte damage and inflammation, as well as marked collagen deposition alongside elevation of fibrosis-associated proteins α-SMA, COL3A1, and fibronectin. Aligned with the existing body of evidence, these findings reinforce the notion of ISO’s profibrotic activity and its chronic heart failure resemblance in experimental paradigms.

In the present study, Pelargonidin was administered at two dosage levels, 20 mg/kg/day (low dose) and 40 mg/kg/day (high dose), due to its previously established efficacy and safety profile in rodent models. A pharmacokinetic study which demonstrated detectable plasma levels of Pelargonidin’s glucuronide metabolite with peak concentrations in 1–2 h and clearance by 18 h in mice which supports the concept of daily dosing schedules [30]. Accordingly, the objective was to study the dose-dependent effects of Pelargonidin on ISO-induced myocardial fibrosis and the alteration of the TGF﷓β/Smad2/3 signaling pathways as well as Th2 cytokine responses with emphasis on the previously mentioned dosages. Interestingly, Pelargonidin dose-dependently mitigated ISO-induced cardiac injury and fibrosis. Marked reduction of serum CK and LDH levels, indicative of robust cardiomyocyte injury mitigation, was observed in the high-dose Pelargonidin group (40 mg/kg/day). Additionally, histopathological and biochemical analyses demonstrated reversal of collagen deposition, hydroxyproline content was lower and collagen I staining was less intense in cardiac tissues. These data confirm the antifibrotic effects of Pelargonidin and its ability to treat myocardial fibrosis.

Pelargonidin’s antifibrotic activities in treating myocardial fibrosis may be linked to at least in part the suppression of the TGF-β1/Smad signaling pathway. TGF-β is well recognized to exacerbate fibrosis in several organs, including the heart [31]. It drives fibroblast activation, myofibroblast differentiation, and ECM protein synthesis, such as collagen and fibronectin. The downstream profibrotic effect is mediated by canonical Smad signaling, which is based on phosphorylation and the nuclear translocation of Smad2 and Smad3. In ISO-treated mice, we noticed marked upregulation of TGF-β1 along with heightened phosphorylation of Smad2 and Smad3. These alterations were significantly reversed after Pelargonidin treatment, showing that Pelargonidin inhibits TGF-β-driven fibrogenic signaling. More significant results in this regard were seen in the high-dose group, which corresponded with the changes observed in histological analysis.

Additionally, Pelargonidin suppressed the expression of important ECM remodeling genes like MMP-9, TIMP1, and elastin. MMPs and TIMPs are responsible for the maintenance of ECM homeostasis, and their imbalance is critical for the development of pathological ECM accumulation and fibrosis [32,33]. ISO increased the expression of MMP-9 and TIMP1, which suggested an actively progressing maladaptive ECM remodeling response. The observation that Pelargonidin normalized the expression of these genes indicates that it mitigates the imbalance between ECM production and degradation, thereby reinforcing its antifibrotic action. The decrease in elastin, which is another component of the structural ECM, also suggests that the myocardial matrix is restored to normal under the influence of Pelargonidin.

Besides the effects on fibrotic signaling, Pelargonidin caused notable immunomodulatory effects, especially through weakening the Th2 cytokine response. ISO treatment significantly increased Th2 hallmark cytokines, IL-4 and IL-13. These cytokines, as increasingly acknowledged, may be important in the fibrogenesis process. IL-4 and IL-13, along with fibroblast proliferation, collagen synthesis, and TGF-β activity, increase the profibrotic effects [34]. The marked reduction of IL-4 and IL-13 by pelargonidin indicates its ability to disrupt the pathological feedback loop between Th2 cytokines and TGF-β signaling. Normally, IL-4 and IL-13 promote TGF-β activation, driving fibroblast proliferation and ECM deposition, while TGF-β further enhances Th2 differentiation and cytokine release [34]. By lowering these cytokines, pelargonidin breaks this cycle, thereby reducing fibroblast activation and halting progressive cardiac fibrosis and remodeling. The modulatory effect of pelargonidin on the Th2 cytokine response may occur through its ability to inhibit key signaling pathways involved in Th2 differentiation and activation, particularly the STAT6/GATA3 axis, which drives the transcription of IL-4 and IL-13 [35]. By suppressing STAT6 phosphorylation and GATA3 expression, pelargonidin likely impedes the maturation and expansion of Th2 cells, thereby reducing their secretion of profibrotic cytokines. Additionally, pelargonidin’s antioxidant properties help mitigate oxidative stress, a known enhancer of Th2 immune responses, while its anti-inflammatory action may downregulate upstream mediators such as NF-κB, further dampening Th2 cytokine production [36]. Through these mechanisms, pelargonidin exerts a dual effect by limiting Th2-driven inflammation and disrupting the positive feedback loop between Th2 cytokines and TGF-β signaling, ultimately contributing to the attenuation of myocardial fibrosis.

Higher antifibrotic capabilities were observed with Pelargonidin compared to Captopril, the control drug in this study and a well-known angiotensin-converting enzyme (ACE) inhibitor. Unlike Pelargonidin, captopril’s benefits stem mostly from RAS inhibition and known effects on TGF-β expression [37]. Captopril and Pelargonidin’s mechanisms are distinct; the former being broader anti-inflammatory and antioxidant actions. This means that Pelargonidin has the potential to supplement existing standard-of-care medications or serve as a botanical alternative with lower side effects. Although the precise molecular interactions of Pelargonidin with fibrosis-related proteins such as TGF-β or MMPs were not directly evaluated in this study, its observed inhibitory effects on TGF-β/Smad2/3 signaling and Th2 cytokines suggest that Pelargonidin may modulate these key fibrotic pathways. Future studies, including molecular docking or *in vitro* binding assays, could help elucidate the direct interactions underlying its anti-fibrotic effects.

## Conclusions

In conclusion, our investigation reveals that pelargonidin significantly inhibits isoproterenol-induced myocardial fibrosis in mice. This protective effect seems to partially involve the inhibition of the TGF-β/Smad2/3 signaling pathway as well as the downregulation of some Th2 cytokines, IL-4 and IL-13. By reducing collagen deposition and modulating key fibrotic and inflammatory markers, pelargonidin exhibits potent anti-fibrotic properties The data indicates that pelargonidin could be a valuable pharmacological option in the prevention and management of myocardial fibrosis and perhaps other fibrotic diseases of the cardiovascular system. However, these findings are limited by species differences, the relatively short study duration, and the absence of pharmacokinetic evaluation. Further research, particularly clinical studies, is necessary to establish its human efficacy and safety profile.

## Figures and Tables

**Table 1:** Primer sequences used for qRT-PCR analysis.

*Gene name*	Sequence (5′-3′)
*MMP9*	FP: CATTCGCGTGGATAAGGA RP: ACAAGAAAGGACGGTGGG
*TIMP1*	FP: GCAACTCGGACCTGGTCATAA RP: CGGCCCGTGATGAGAAACT
*Elastin*	FP: CAGCTAAATACGGTGCTGCTG RP: AATCCGAAGCCAGGTCTTG
*β-actin*	FP: TTGCAGCTCCTTCGTTGCC RP: GACCCATTCCCACCATCACA
